# Recent South African court rulings on failing to disclose HIV status to sexual partners

**DOI:** 10.4102/safp.v66i1.6014

**Published:** 2024-12-17

**Authors:** Jillian B. Gardner, Siraaj Khan

**Affiliations:** 1 Steve Biko Centre for Bioethics, Faculty of Health Sciences, University of the Witwatersrand, Johannesburg, South Africa

**Keywords:** criminalisation, criminal liability, disclosure, health professional, HIV, legal obligation, partner notification, sexual transmission

## Abstract

In South Africa, the sexual transmission of human immunodeficiency virus (HIV) between partners is criminalised under general criminal law offences such as attempted murder, assault and rape, despite the absence of specific HIV-related legislation. Recent court cases have opened the door to securing convictions.

**Contribution:**

These cases highlight the serious legal consequences of non-disclosure and the importance of consent to sexual intercourse. Health professionals in South Africa have a legal and ethical duty to counsel patients on HIV and may be obligated to inform patients who refuse to disclose their status to their partners about the potential legal implications under South African criminal law.

## Introduction

In 2022, it was estimated that there were around 39 million people in the world living with human immunodeficiency virus (HIV): 37.5 million adults and 1.5 million children under the age of 15 years.^1,2^ There were roughly 630 000 acquired immunodeficiency syndrome (AIDS)-related deaths and approximately 1.3 million new infections per day.^1^ South Africa bears the world’s heaviest burden of HIV and/or AIDS, with nearly 8 million people living with HIV, accounting for almost one-fifth of all global HIV cases. Approximately 14% of the population is HIV-positive, and among adults aged 15–49 years, the estimated prevalence is 17.8%.^3^ Significant progress has been made in the fight against HIV and/or AIDS, including the expansion of HIV testing, rollout of antiretroviral therapy (ART) and implementation of prevention programmes, which have dramatically changed the face of HIV and/or AIDS from life-threatening to manageable. However, it remains incurable and fatal over time if not managed.

Despite increased efforts over time to enable more people to learn about their HIV status globally, as many as one in six people infected with HIV are still unaware of their being infected.^1,4^ In 2022, only 86% of people living with HIV knew their HIV status, 76% were accessing treatment, and 71% were virally suppressed.^1^ Even though most people aged 15 years and older living with HIV in South Africa are aware of their status, a sizeable number are unaware that they are living with HIV.^3,4,5,6,7^ Globally, and including South Africa, the primary mode of HIV transmission is through unprotected sexual contact, including vaginal and anal sex.^8,9,10^ Disclosing HIV status to sexual partners has the potential to decrease the likelihood of transmitting the virus through sexual contact.^11,12^ The lack of disclosure by individuals about their HIV status to their sexual partners has sparked discussions regarding the criminalisation of behaviours, such as not informing sexual partners about their HIV status and potentially infecting them with the virus.

The laws and policies that penalise individuals living with HIV for behaviours that could potentially expose others to the virus are intended to discourage behaviours that might lead to transmission.^13,14^ These laws vary widely across jurisdictions. Some countries use existing laws to prosecute individuals for HIV transmission or exposure, charging them for crimes such as murder, manslaughter and assault.^15,16,17^ Others have specific HIV-related laws.^18,19^ The South African Law Reform Commission (SALC) has recommended that existing common law is sufficient to address the sexual transmission of HIV between sexual partners and that specific statutory intervention is unnecessary.^20^ By default, these actions are criminalised in South Africa.

This article discusses the implications of recent judicial decisions that have shaped the South African legal landscape regarding the transmission of HIV among sexual partners. The Phiri^21,22^ and Conga^23,24^ cases (discussed later in this article) illustrate how the general principles of criminal law have been applied in South Africa to address HIV transmission and exposure between sexual partners in the absence of specific HIV-related legislation. These cases underscore the legal responsibility of individuals living with HIV to disclose their status to sexual partners. Failure to do so may result in severe legal consequences including criminal charges. These cases also imply that health professionals in South Africa may be obligated to explicitly inform patients who refuse to disclose their HIV status to their sexual partners that they may be prosecuted under South African criminal law. The authors posit that health professionals may lack awareness of their legal obligations or the potential legal ramifications of HIV transmission through sexual contact. This deficiency in knowledge may be attributed to various factors, including the critical shortage of medical practitioners in the country.^25,26^ Additionally, discrepancies may exist between patients’ and doctors’ expectations regarding the initiation of discussions about sexual difficulties, as well as their respective relational and clinical priorities during consultation.^27^

Increasing legal literacy among health professionals is crucial to ensure that they understand the potential legal implications of non-disclosure and adequately inform their patients.^28^

## Current South African law governing the transmission of HIV between sexual partners

No specific laws exist in South Africa that expressly make it an offence to withhold, transmit or reveal HIV status to a consenting adult partner during sexual activity.^22^ This approach reflects a broader legal and public health strategy that focusses on the use of existing legal frameworks rather than on HIV-specific criminal legislation. Cases of HIV transmission and exposure have been prosecuted under general criminal laws such as attempted murder, assault and rape. Two cases were recently brought before the courts where the accused was charged with attempted murder and rape.

In Phiri vs. S.,^21,22^ a former Department of Health HIV counsellor was convicted of attempted murder for knowingly infecting his partner with HIV. Phiri was living with HIV for 3 years prior to meeting the appellant, and although he was aware of his status, he did not disclose this to her. The appellant testified that Phiri had declined to use a condom when she requested him to do so. In the Conga^23,24^ case, a former member of the South African National Defence Force was convicted of attempted murder and rape for failing to disclose his HIV status and knowingly infecting his former girlfriend with HIV.^23,24^ The complainant:

[*T*]estified that he would remove the condom during sexual intercourse, despite the fact that they on those occasions agreed that they would only have intercourse if he wore a condom and he also on various occasions assured her that he was HIV-negative. Furthermore … that the accused also refused to go for an HIV test with her and became angry if she would ask him about his HIV status.^29^

These cases contribute to the legal landscape in South Africa regarding the sexual transmission of HIV between sexual partners. They highlight how the principles of criminal law have been used to prosecute the sexual transmission of HIV, and consequently, the serious legal implications of non-disclosure and the sexual transmission of HIV among sexual partners. They set a significant legal precedent, reinforcing the criminal liability of individuals who knowingly expose others to HIV, without disclosure. These cases highlight the importance of consent in sexual relationships and the legal responsibility of individuals to disclose their HIV status to their sexual partners.

## Implications for health professionals

Health professionals play a critical role in educating patients about HIV and preventing its transmission. Health professionals have a legal and ethical duty to counsel patients on HIV and offer HIV testing.^30,31^ Health professionals have an ethical duty to counsel patients on the importance of disclosure and safe practices. They must balance confidentiality with the need to protect public health. They play a crucial role in educating patients about their responsibilities and the potential consequences of their actions;^17^ thus, they must provide counselling to patients about the implications of non-disclosure and unsafe sexual behaviours.

In South Africa, health professionals have a general duty to maintain patient confidentiality, including the HIV status of their patients.^32^ This means that they may not disclose a patient’s HIV status unless the patient consents to the disclosure or if specific legal exceptions apply. The Health Professions Council of South Africa (HPCSA) guidelines emphasise the importance of maintaining confidentiality.^30^ Disclosure without consent may be justified if it is in the public interest, such as when there is a significant risk to others, or if it is mandated by court order or specific legislation.^32^

Health professionals may face legal and ethical dilemmas when dealing with patients who refuse to disclose their HIV status to their sexual partners.^14^ While maintaining patient confidentiality, health professionals should encourage patients to disclose their status voluntarily. Failure to provide adequate counselling and information could result in disciplinary action by the medical regulatory bodies. Guidelines from the HPCSA and other bodies in South Africa emphasise the importance of confidentiality, informed consent and the ethical duty to provide comprehensive counselling.^28^ According to the HPCSA, ethical guidelines for good practice in the healthcare professions are:

9.1 Health care practitioners should try to encourage their HIV-positive patients to disclose their status to their sexual partners so as to encourage them to undergo VCT and access treatment if necessary. This is consistent with good clinical practice.

9.2 If the patient refuses consent, the health care practitioner should use his or her discretion when deciding whether or not to divulge the information to the patient’s sexual partner, taking into account the possible risk of HIV infection to the sexual partner and the risks to the patient (e.g. through violence) that may follow such disclosure. The decision must be made with great care, and consideration must be given to the rights of all the parties concerned. If the health care practitioner decides to make the disclosure against the patient’s wishes, the practitioner must do so after explaining the situation to the patient and accepting full responsibility at all times. The following steps are recommended – the health care practitioner must:

9.2.1 Counsel the patient on the importance of disclosing to his or her sexual partner and on taking other measures to prevent HIV transmission.

9.2.2 Provide support to the patient to make the disclosure.

9.2.3 If the patient still refuses to disclose his or her HIV status or refuses to consider other measures to prevent infection, counsel the patient on the health care practitioner’s ethical obligation to disclose such information.

9.2.4. If the patient still refuses, disclose information on the patient’s HIV status to the sexual partner and assist them to undergo VCT and access treatment if necessary.

9.2.5 After disclosure, follow up with the patient and the patient’s partner to see if disclosure has resulted in adverse consequences or violence for the patient, and, if so, intervene to assist the patient appropriately.

9.3 Health care practitioners must recognise the major ethical dilemma when confronted with a person who is HIV-positive and who refuses, despite counselling, to inform his/her partner or partners.^31^

Health professionals must adhere to professional standards and guidelines. Notably, the HPCSA places responsibility on health professionals to exercise their discretion in deciding to disclose. This raises the question of whether it is fair to transfer what is primarily the patient’s responsibility to disclose to health professionals. There is an ongoing debate on whether health professionals should breach confidentiality to inform at-risk individuals, balancing the duty to protect public health with the duty to maintain patient trust. Should there be sanctions for health professionals in the event that they fail to inform patients about the legal consequences of non-disclosure? The role of health professionals in advising patients about legal risks has been debated, with some arguing that it extends beyond traditional medical advice.^33,34^

In the Phiri^21,22^ and Conga^23,24^ cases, the courts acknowledged that there is a general legal duty in South Africa to disclose one’s HIV-positive status to a sexual partner before engaging in sexual activity. Health professionals must adhere to the ethical guidelines set by the HPCSA. If a health professional fails to inform a patient about the risk of HIV transmission and the legal implications of non-disclosure, and if this omission leads to harm, they could potentially be found guilty. Proper documentation of informed consent and counselling sessions is crucial to protect health professionals from potential liability. The HPCSA advises health professionals to use their discretion when deciding whether to inform a patient’s partner about their HIV status, considering the risks to both the patient and partner. However, it may be difficult to exercise this discretion. Thus, the aforementioned case law may assist health professionals in this regard.

## Conclusion

Legal cases can exacerbate stigma against people living with HIV, making it more challenging for them to disclose their status and seek support.^14^ The fear of criminal prosecution may deter individuals from being tested or disclosing their status, which can negatively affect public health. Educating the public about the legal implications of non-disclosure and sexual transmission of HIV can help individuals understand their responsibilities and the potential consequences of their actions. Promoting considerations such as the importance of consent to sex and mutual respect in relationships can foster a culture of responsibility and care.^14,17^

While health professionals in South Africa are generally required to maintain patient confidentiality, they must also navigate complex ethical and legal considerations when it comes to disclosure of HIV status. They can be held liable for negligence if they fail to provide adequate information and counselling to their patients about the legal implications of non-disclosure. While there is no explicit legal requirement for health professionals to inform patients about the specific criminal charges for non-disclosure of HIV status, failing to provide comprehensive information could have legal and ethical repercussions.
